# Group 13 ion coordination to pyridyl breaks the reduction potential *vs.* hydricity scaling relationship for dihydropyridinates†

**DOI:** 10.1039/d3sc03806h

**Published:** 2023-11-16

**Authors:** Leo W. T. Parsons, James C. Fettinger, Louise A. Berben

**Affiliations:** a Department of Chemistry, University of California Davis CA 95616 USA laberben@ucdavis.edu

## Abstract

The relationship *E*_p_*vs.* Δ*G*_H–_ correlates the applied potential (*E*_p_) needed to drive organohydride formation with the strength of the hydride donor that is formed: in the absence of kinetic effects *E*_p_*vs.* Δ*G*_H–_ should be linear but it would be more energy efficient if *E*_p_ could be shifted anodically using kinetic effects. Biological hydride transfers (HT) performed by cofactors including NADH and lactate racemase do occur at low potentials and functional modeling of those processes could lead to low energy HT reactions in electrosynthesis and to accurate models for cofactor chemistry. Herein we probe the influence of *N*-alkylation or *N*-metallation on Δ*G*_H–_ for dihydropyridinates (DHP^−^) and on *E*_p_ of the DHP^−^ precursors. We synthesized a series of DHP^−^ complexes of the form (pz_2_^H^P^−^)E *via* hydride transfer from their respective [(pz_2_P)E]^+^ forms where E = AlCl_2_^+^, GaCl_2_^+^ or Me^+^. Relative Δ*G*_H–_ for the (pz_2_^H^P^−^)E series all fall within 1 kcal mol^−1^, and Δ*G*_H–_ for (pz_2_^H^P)CH_3_ was approximated as 47.5 ± 2.5 kcal mol^−1^ in MeCN solution. Plots of *E*_p_*vs.* Δ*G*_H–_ including [(pz_2_P)E]^+^ suggest kinetic effects shift *E*_p_ anodically by ∼215 mV.

## Abstract 2

The relationship *E*_*p*_*vs. ΔG*_H_− correlates the applied potential (*E*_*p*_) needed to drive organohydride formation with the strength of the hydride donor that is formed: hydride transfer catalysis - as in enzymes like LarA - will be more energy efficient if *E*_*p*_ is shifted anodically using kinetic effect.

## Graphical abstract

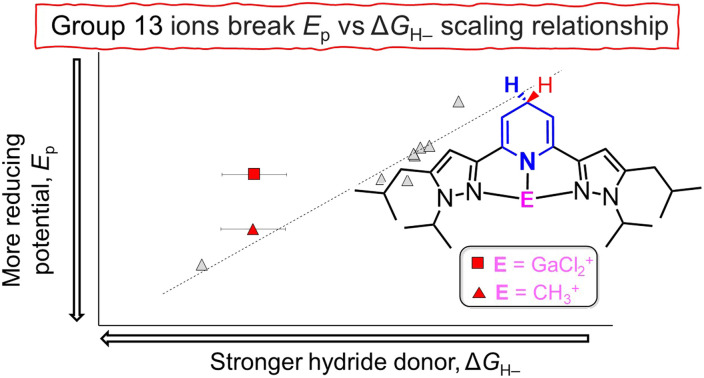

## Introduction

Reduction reactions involving hydride transfer (HT, formally two electrons and a proton) are important in transformations ranging from CO_2_ reduction^1,2^ to selective reductions of carbonyls, imines, and alkenes.^3–5^ In enzymes, tremendous selectivity and efficiency in reduction reactions involving HT is achieved by cofactors such as NAD^+^/NADH and the nickel pincer nucleotide (NPN) cofactor in lactate racemase, in a carefully tailored environment.^6,7^ Synthetic chemists have harnessed this control in systems which incorporate enzymes for highly efficient reactions in the presence of catalytic NAD^+^,^8,9^ and synthetic chemists aspire to similar reaction control as in the asymmetric co-catalysis of enantioselective organocatalytic hydride reduction.^10,11^ Alongside these reaction conditions, a wide array of both metal hydride (M–H) and organohydride (C–H) transfer agents has been developed, and ongoing efforts have various directions: improved selectivity for desired products,^12–14^ and functional group tolerance,^15,16^ are just two examples.

**Scheme 1:**
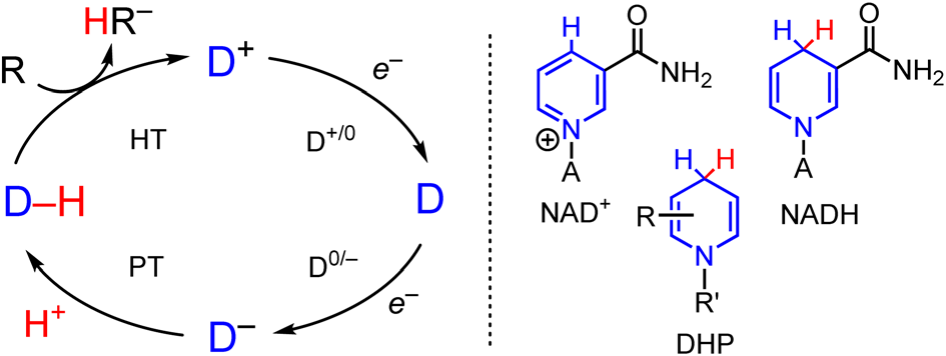
(Left) Outline of electron and proton steps to generic cationic hydride precursor, D^+^, leading to hydride formation of a general hydride donor D–H and subsequent HT to substrate. (Right) Line drawing of NAD^+^, NADH and dihydropyridinate, DHP. A = Adenine dinucleotide.

In the growing area of electrosynthesis and electrocatalysis additional considerations need to be made for the design of HT reagents that can be effectively (re)generated electrochemically. These include choice of proton source for hydride regeneration and catalytic turnover and design of precursors to the hydride which lower the applied potential required for the reduction steps that (re)generate the hydride reagent in the catalytic cycle (Scheme 1).^17–19^ The lowered potential is needed to save energy, and minimize side reactions including proton reduction to H_2_ and decomposition of catalyst or substrate. It is well-known that the half wave potential, *E*_1/2_(D^+/0^) for the hydride precursor (D^+^ in Scheme 1) scales with the free energy change associated with loss of hydride ion from a metal- or organo-hydride as in eqn (1):^20,21^

1 D–H → D^+^ + H^−^ free energy: Δ*G*_H–_

This free energy change is also called hydricity (Δ*G*_H–_). Experimentally, hydricity is determined using thermochemical cycles that are well described in the literature,^22,23^ and cycles relevant to this work are included below (*vide infra*).

Hydricity can be a useful predictor for the overall driving force for an HT reaction and influence reaction selectivity, where a smaller Δ*G*_H–_ value indicates a stronger thermodynamic driving force for HT, *i.e.* a stronger hydride donor. For example, electrocatalytic reduction of CO_2_ to HCOO^−^ is optimized when the hydride donor is sufficiently hydridic to make CO_2_ reduction thermodynamically favorable but not so strong as to undergo competitive H_2_ evolution *via* reaction of the hydride with protons.^24–27^ Both *E*_1/2_ and Δ*G*_H–_ are intrinsic thermodynamic properties of the hydride donor reagent.^28,29^ In practice, reduction of D^+^ is often coupled with proton transfer (PT) reactions and then the CV waveform manifests as an irreversible redox event where both thermodynamic and kinetic contributions to the D^+/0^ and D^0/−^ redox couples and PT influence the necessary applied potential (*E*_p_) that is required to generate D–H.^30^ Metal ion coordination is known qualitatively to lower *E*_p_ for organic molecules including DHP^−^s but not enough data has been reported to draw any correlations between structure and function.^31,32^

**Scheme 2:**
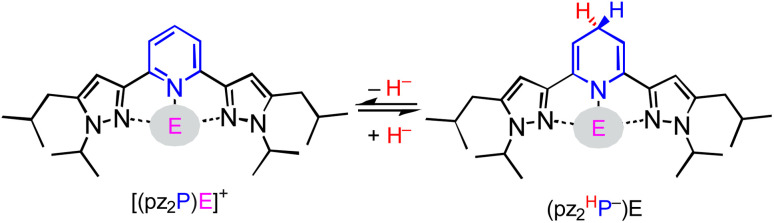
Interconversion of (pz_2_P)E^+^ with (pz_2_HP^−^)E *via* HT, where E = AlCl_2_^+^, GaCl_2_^+^, and CH_3_^+^.

In this report, we investigate structural tuning of dihydropyridinates (DHP^−^) and their precursors *via* metal ion effects. To assess the results of metal ion coordination we considered the scaling relationship between *E*_p_*vs.* Δ*G*_H–_ using extensive data reported in the literature, and as discussed below.^21,33,36^ The dipyrazolylpyridine ligand platform (pz_2_P) supports complexes with the form [(pz_2_P)E]^+^ where E = GaCl_2_^+^, CH_3_^+^ and AlCl_2_^+^, and those compounds are denoted herein as 1^+^, 2^+^ and 4^+^ respectively.^30,36^ In this report the syntheses of organohydrides (pz_2_^H^P^−^)E, are reported for E = GaCl_2_^+^ and CH_3_^+^, *via* a formal addition of hydride (two electrons and one proton) to [(pz_2_P)E]^+^ (Scheme 2). Characterization of the geometric and electronic structures of 1H, 2H and 4H was performed and the hydride donor ability for each compound benchmarked. A comparison of the properties of 1H and 2H with those of previously reported organohydride reagents reveals reduction potential is lowered by + 215 mV for 1H and does not deviate within error for 2H relative to *E*_p_ expected according to the *E*_p_*vs.* Δ*G*_H–_ scaling relationship. The origin of this effect is discussed.

## Results and discussion

### Preparation of compounds

**Scheme 3:**
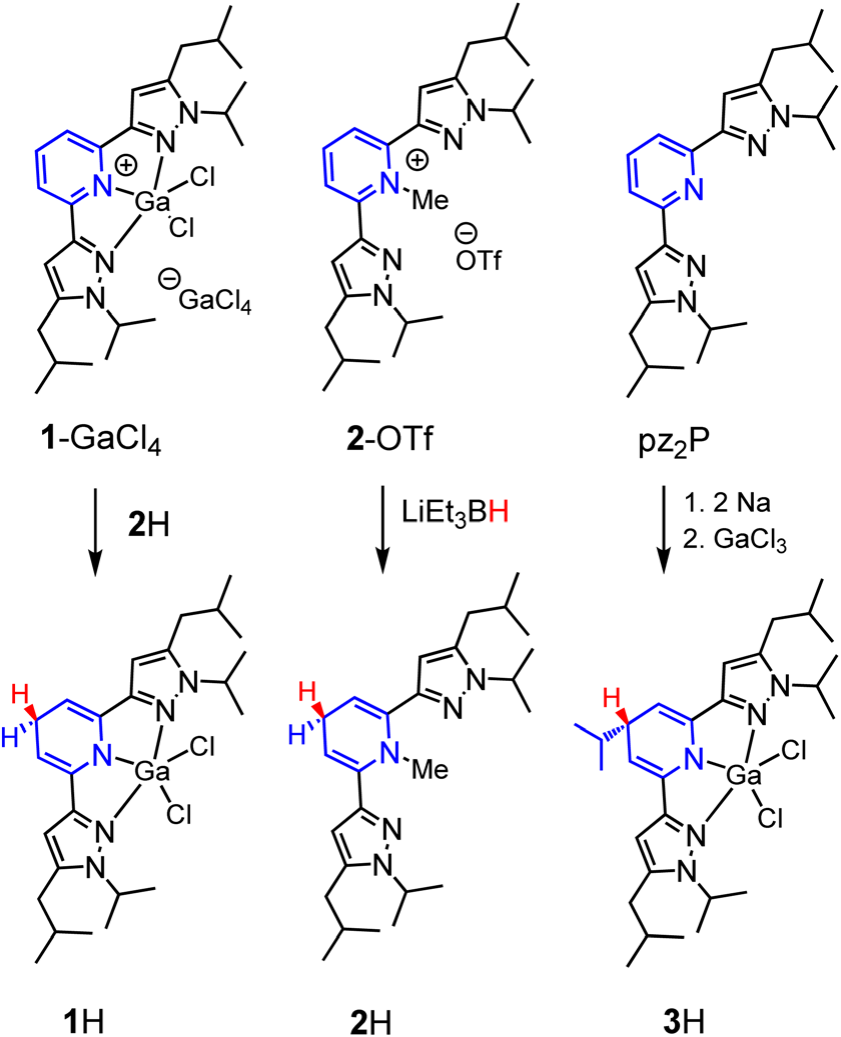
Syntheses of 1H, 2H and 3H.

**Fig. 1:**
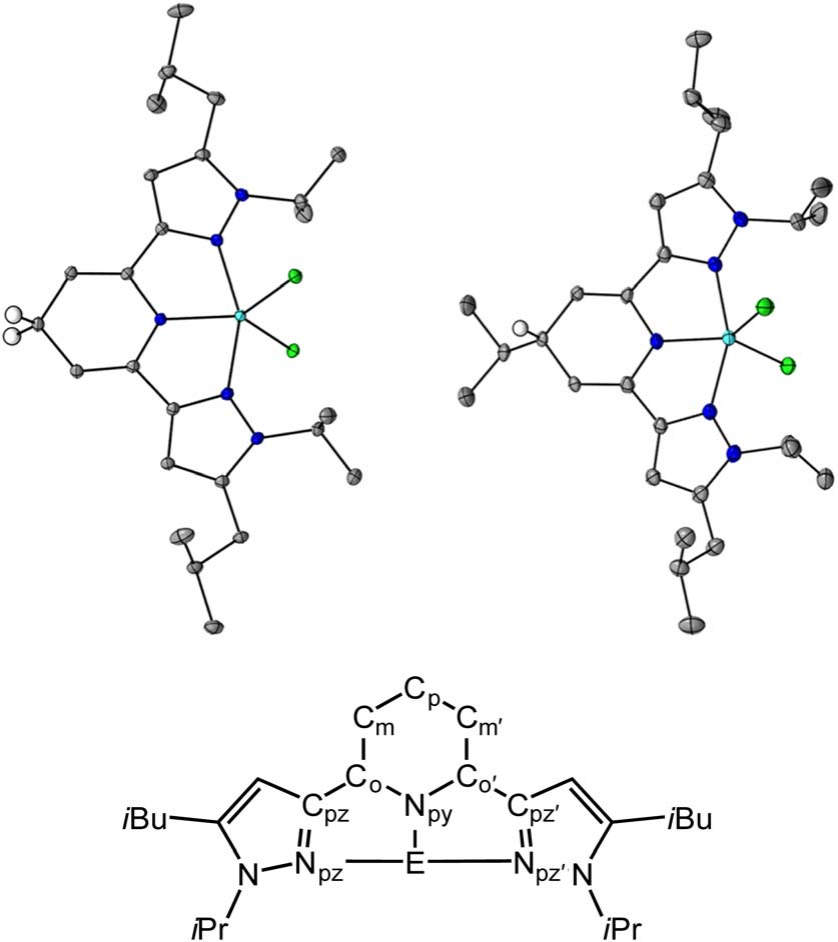
Solid state structures of 1H (top left) and 3H (top right) and pz_2_P atom naming convention used throughout (bottom). Blue, light blue, green, gray ellipsoids, and white circles represent N, Ga, Cl, C, and H atoms, respectively. H atoms except C_p_–H omitted for clarity. The thermal ellipsoids are shown at 30% probability.

We targeted the 1,4-DHP^−^‘s derived from [(pz_2_P)GaCl_2_]^+^ (1^+^) and [(pz_2_P)CH_3_]^+^ (2^+^) (Scheme 3). In an attempt to generate [(pz_2_^H^P^−^)]GaCl_2_ (1H), two equivalents of Na were added to a solution of one equivalent pz_2_P in THF at room temperature and the clear solution turned deep red following consumption of the Na metal over 24 h.^35^ Addition of one equivalent of GaCl_3_ resulted in a further colour change to dark purple. The ^1^H NMR spectrum of the isolated product showed no resonances corresponding to protons on an aromatic pyridine ring but instead resonances at 5.21 and 3.57 ppm were observed. These resonances are consistent with a 1,4-DHP^−^ ligand form coordinated to GaCl_2_^+^, and consistent with our previous report of [(pz_2_^H^P^−^)]AlCl_2_, 4H,^36^ where the resonances were observed at 4.99 and 3.50 ppm. However, integration of the resonance at 3.57 ppm gives a value of 1H atom for the C_p_–H proton (see Fig. 1 for carbon atom labelling scheme), but integration of 2H atoms is expected for formation of 1H. A multiplet at 1.76 and doublet at 1.12 ppm corresponding to integrations of 1H atom and 6H atoms, respectively, suggest substitution of an *i*Pr group at the C_p_ carbon (Fig. S1†), and this was confirmed by single crystal X-ray diffraction which identified [(pz_2_^*i*Pr^HP^−^)]GaCl_2_ (3H), (*vide infra*). Presumably, a reductive cleavage of *N*-isopropyl generates isopropylsodium, which, in turn, reacts with the pyridine ring to form 3H.^37^

### Synthesis of 1H was also attempted *via* several additional routes

Reduction of [(pz_2_P)GaCl_2_]GaCl_4_ (1-GaCl_4_) was attempted *via* reactions with sodium metal, sodium napthalenide, and decamethylcobaltocene, but these reactions did not yield clean isolable products. Reactions of 1-GaCl_4_ with hydride donors NaBH_4_, LiAlH_4_, and LiEt_3_BH also did not yield 1H. Ultimately, we obtained 1H in 55% yield as crystals formed from a concentrated solution of 1-GaCl_4_ and 2H in MeCN (the synthesis of 2H is described in the next paragraph). The ^1^H NMR spectrum of 1H showed no aromatic resonances, but the C_p_–H resonance is observed as a triplet at 3.48 ppm, and this is consistent with DHP^−^ formation as observed in 4H, 2H, and 3H which have C_p_–H resonances at 3.50, 3.62 and 3.57 ppm, respectively. The pyrazole ring proton resonance is a singlet, shifted upfield, from 7.01 in 1^+^ to 5.92 ppm in 1H (Fig. S2†). The composition of 1H was further confirmed by X-ray diffraction and combustion analysis.

The synthesis of 1,4-DHP^−^s from *N*-alkylated pyridinium salts has been reported, using sodium dithionate,^38^ or a metal hydride donor as reductant.^39^ For the preparation of 2H we found that addition of 1.3 equivalents of lithium triethyl borohydride, LiEt_3_BH, to a colourless solution of 2-OTf in THF resulted in an instant colour change to a yellow solution. After stirring for 5 minutes the solution was concentrated to a residue which was dissolved in hexane and filtered. The yellow oily filtrate yielded pz_2_(*N*-Me^H^P), 2H, in 27% yield following workup. The proton NMR spectrum of 2H is consistent with the 1,4-DHP^−^ structure and with NMR resonances at 5.62 and 3.24 ppm (Fig. S3†). The composition of 2H was confirmed using HRMS.

### Solid state structures

Crystals suitable for single crystal X-ray diffraction of the 1,4-DHP^−^ ligand compounds, 1H and 3H, were obtained from saturated solutions of MeCN and hexane respectively over a period of three days, and were obtained as purple, and colorless block shaped crystals, respectively (Tables S1 and S2,†Fig. 1). We were not able to obtain crystals of 2H despite many attempts. For 1H, the average bond lengths of the N_py_–C_o_, C_o_–C_m_ and C_p_–C_m_ bonds are 1.387(6), 1.343(7) and 1.507(7) Å, respectively, and for 3H these are 1.433(6), 1.33(2) and 1.507(8) Å, respectively (see Fig. 1 for pz_2_P atom naming). The increased bond lengths for C_p_–C_m_ in both 1H and 3H relative to 1^+^, are characteristic of 1,4-DHP^−^ structures. Upon formation of the 1,4-DHP^−^, the geometry around the Ga(iii) center becomes closer to trigonal bipyramidal with a *τ*_5_ value of 0.76 for both 1H and 3H.^40^ There is also an increase in the N_pz_–Ga–N_pz’_ bond angle from 154.9(1)° in 1^+^ to 156.47(3)° and 156.51(8)° in 1H and 3H respectively.^30^ The ligand twists to accommodate the distorted trigonal bipyramidal geometry as indicated by the torsion angle between the two coordinating pyrazine arms C_pz_=N_pz_C_pz′_=N_pz’_ which increase by 2.89° and 1.91° for 1H and 3H respectively relative to 1^+^. There is no significant difference in the bond distances within the pyrazole rings between all compounds discussed (Tables S3 and S4†).

### Hydride transfer reactions of 1H, 2H and 4H

There are several possible experiments that can be used to obtain Δ*G*_H-_.^22^ We found hydride transfer best suited for 1H, 2H and 4H where chemical equilibria were established between 1H, 2H and 4H, and organohydride molecules of known Δ*G*_H-_. Using this method, direct HT from a donor (D–H) to acceptor (A^+^) is observed and then an equilibrium constant can be determined which establishes the difference in Δ*G*_H-_ between the donor and acceptor. The relationship between the hydride donor ability of D–H and A–H, and the hydride transfer between D^+^ and A^+^ is described by eqn (2)–(5):^22^

2 D–H + A^+^ ⇌ D^+^ + A–H *K*_(2)_

3 A–H ⇌ A^+^ + H^−^ Δ*G*_H−_(A–H)

4 D–H ⇌ D^+^ + H^−^ Δ*G*_H–_(D–H)

where Δ*G*_H–(4)_ = Δ*G*_H–(2)_ – Δ*G*_H–(3)_

and

5 Δ*G*_H–(2)_ = –*RT* ln(*K*_(2)_)

**Chart 1:**
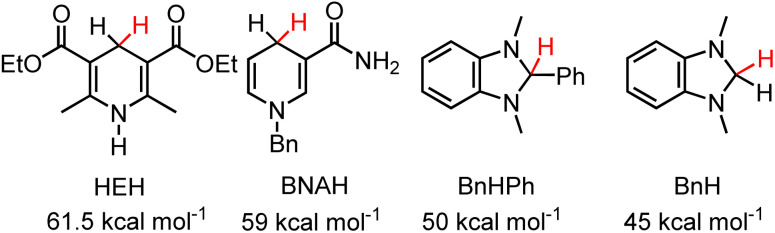
1,4-DHPs and benzimidazoles discussed in the text and their reported hydricities in MeCN.^21^

We first set out to determine the hydricity of 2H, because 2H is the easiest of the hydrides to make, with a series of hydride acceptors of known hydricity. Reaction of 2-OTf with one equivalent of 1,3-dimethyl-2-phenyl-2,3-dihydro-1*H*-benzo[*d*]imidazole, BnHPh (Δ*G*_H–_ = 50 kcal mol^−1^ in MeCN, (Chart 1))^19^ resulted in HT which was observed by ^1^H NMR in CD_3_CN after 2 days and approached equilibrium after two weeks, as in eqn (6):

6 BnHPh + 2^+^ ⇌ BnPh^+^ + 2H

7 Bn^+^ + 2H ⇌ BnH + 2^+^

HT from 2H to one equivalent of 1,3-dimethyl-1*H*-benzimidazolium, Bn^+^ (Δ*G*_H–_ = 45 kcal mol^−1^ in MeCN (Chart 1))^29^ was also observed by ^1^H NMR after 3 days and approached equilibrium after two weeks (eqn (7), Fig. S4 and S5†). The equilibria were heavily reactant favoured suggesting that the hydricity of 2H lies close to the middle of the range from 45–50 kcal mol^−1^ in MeCN solution. Control experiments run in parallel consisting of the starting materials 2-OTf, 2H, 1,3-dimethyl-2-phenyl-2,3-dihydro-1*H*-benzo[*d*]imidazole and 1,3-dimethyl-1*H*-benzimidazolium iodide in CD_3_CN with trimethoxybenzene as an internal standard show no changes after 2 weeks (Fig. S6–S9†). We, therefore, estimate for 2H that Δ*G*_H-_ = 47.5 ± 2.5 kcal mol^−1^. These measurements put the Δ*G*_H–_ of 2H on the lower side (stronger hydride donor) of values reported for 1,4-DHPs which have been reported from 73–43 kcal mol^−1^.^21^ As a reference point to commonly employed DHPs, the Hantsch ester (HEH) has Δ*G*_H–_ = 61.5 kcal mol^−1^ and BNAH (1-benzyl-1,4-dihydronicotinamide) has Δ*G*_H–_ = 59 kcal mol^−1^. An established trend is that DHP^−^s are more hydridic when they are functionalized with electron donating groups. Both the Hantzsch ester and BNAH feature electron withdrawing groups (Chart 1), whereas 2H has two moderately electron donating pyrazole groups. The hydricity of 2H is similar to methyl substituted *N*-alkylated DHP^−^s.^21^

**Scheme 4:**
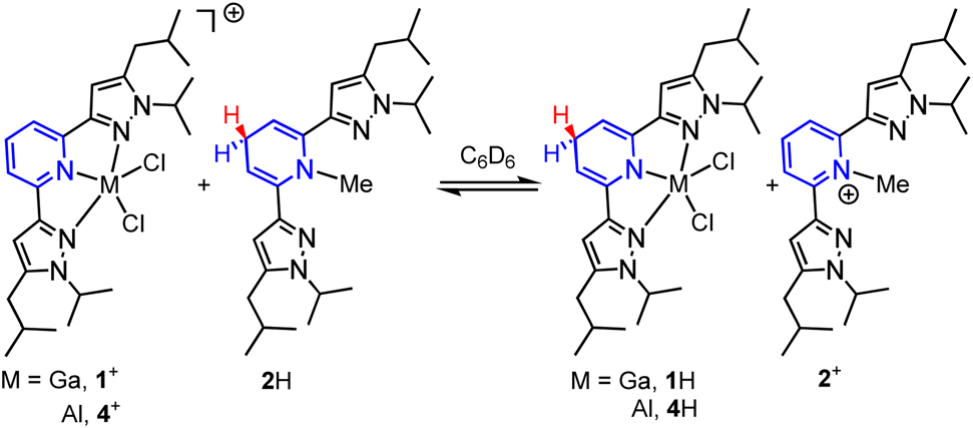
HT equilibria between 1-GaCl_4_ and 2H, and 4-AlCl_4_ and 2H. Counter ions omitted for clarity.

Having established the hydricity of 2H, we endeavoured to determine if HT from 2H would be observed to either 1^+^ or 4^+^. Reactions of a 1 : 1 molar ratio of 1-GaCl_4_ with 2H, and of [(pz_2_P)AlCl_2_]AlCl_4_, 4-AlCl_4,_ with 2H were monitored by ^1^H NMR in C_6_D_6_ in order to determine the reaction equilibria (Scheme 4).^1,41^ The back reaction in these equilibria corresponds to eqn (2) and were used in calculations of Δ*G*_H–_. Integration of ^1^H NMR signals (C_p_–*H* of 4^+^/4H and 1^+^/1H and N_py_–C*H*_3_ of 2^+^/2H) relative to the internal standard trimethoxybenzene showed equilibria were established after 4 days for reaction of 1^+^ and 2H and after 3 days for the reaction of 4^+^ and 2H (see ESI for full experimental details†). We recognize that solvents such as DMSO, MeCN or H_2_O, which have higher dielectric constants and a wealth of reported hydricity data, would be a better choice for monitoring these equilibria,^23^ but 1-GaCl_4_ is insoluble in DMSO, 1H is sparingly soluble in MeCN, and both 4^+^ and 1^+^ are unstable in protic solvents. Equilibrium constants (*K*) were calculated as 0.83 for the back reaction of 1-GaCl_4_ with 2H and 0.24 for the back reaction of 4-AlCl_4_ with 2H. These values correspond to a difference in Δ*G*_H-_ between 1H and 2H of 0.1 ± 0.1 kcal mol^−1^ and a difference in Δ*G*_H-_ between 4H and 2H of 0.8 ± 0.1 kcal mol^−1^. Given that the values of 1H, 2H and 4H were all found to be within 1 kcal mol^−1^ in benzene, which has a dielectric constant over 15 times lower than MeCN, we estimate that the hydricity values of 1H and 4H in MeCN would differ from 2H by no more than 1 kcal mol^−1^.^25,28,42^ Minor differences in Δ*G*_H-_ might arise from the varied Lewis acidity of the cations.

### Metal ion effects on the scaling relationship between *E*_p_ and Δ*G*_H–_ for organohydrides

Linear correlations between hydricity (Δ*G*_H–_) of a transition metal hydride and the first reversible redox couple, *E*_1/2_(D^+/0^) in Scheme 1, of a parent transition metal complex are well established within several classes of metal complexes.^24,43,44^ Concomitant reports by the groups of Kubiak,^20^ and Glusac,^21^ showed that a more general correlation exists across several classes of transition metal and ligand sets, and across structurally diverse organohydrides.

**Fig. 2:**
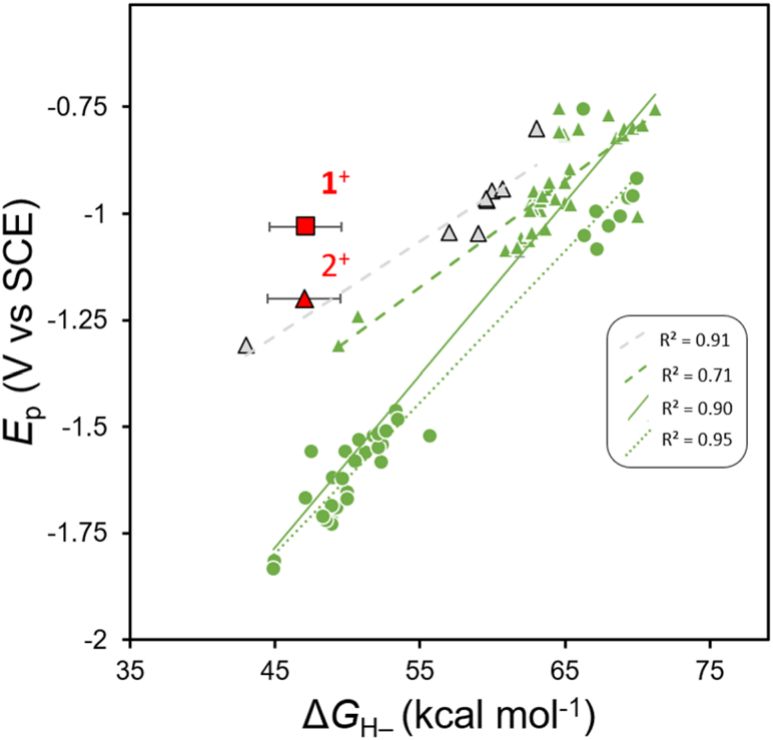
Plot of *E*_p_*vs.* Δ*G*_H_– for benzimidazoles (circles), 1,4-DHPs (triangles) 2^+^ and those previously published and complexe 1^+^. Black outlines around markers represent experimentally determined Δ*G*_H–_ values, green markers indicate computational Δ*G*_H–_ values and red markers corresponds to compounds reported herein. The figure key displays *R*^2^ values for linear fits to: 1,4-DHPs with experimentally determined Δ*G*_H–_ values (dashed grey line).^36,45–48^ 1,4-DHPs with computationally determined Δ*G*_H–_ values (dashed green line),^21,36^ 1,4-DHPs and benzimidazoles with computationally determined Δ*G*_H–_ values (solid green line),^21,33,36^ and benzimidazoles with computationally determined Δ*G*_H–_ values (dotted green line).^21,33^4^+^ is not included on the plot since it is not stable in MeCN.

It is known that many oxidized organohydride precursors display irreversible reduction events, *E*_p_, in CV experiments and we wondered if there is any correlation between a plot of *E*_p_*vs.* Δ*G*_H–_ for those compounds and whether the plot might highlight a variation in kinetic contributions to reduction that are causing the irreversibility. To construct a plot of *E*_p_*vs.* Δ*G*_H–_ we used reported, computationally obtained Δ*G*_H–_ values for *N*-containing heterocyclic organohydrides in MeCN solution,^21^ with their reported irreversible cathodic peak potentials (*E*_p_) obtained using CV in MeCN solution.^33,36^ We compiled *E*_p_ values for various imidazoles,^33^ and substituted 1,4-DHPs;^36^ and all *E*_p_ values were converted to V *vs.* SCE. Linear regression provides a linear relationship (Fig. 2). We notice that when fit independently, predicted *E*_p_ values for 1,4-DHPs are at more positive potentials than benzimidazoles with comparable Δ*G*_H–_ within the reported Δ*G*_H–_ range. Furthermore, another anodic shift in predicted *E*_p_ values is observed from fits of experimental Δ*G*_H–_ values of 1,4-DHPs,^45–48^ compared to those reported from computational methods. Experimentally measured values of Δ*G*_H–_ (for 1H and 2H) and of *E*_p_ (for 1^+^ and 2^+^) were added to the plot of *E*_p_*vs.* Δ*G*_H–_ for comparison (red symbols, Fig. 2). Relative to the correlation line, 1^+^ is more easily reduced: *E*_p_(1^+^) is +215 mV more anodic than predicted. The Al-supported DHP compound 4H has a similar *E*_p_ to 2H when both are measured in THF, and this suggests that both AlCl_2_^+^ and GaCl_2_^+^ similarly break the *E*_p_*vs.* Δ*G*_H–_ scaling relationship, but there is not enough data available in THF to illustrate this point on a plot as we do for MeCN in Fig. 2. All the organohydrides plotted have cationic oxidized forms so the overall charge on the molecules should not be a factor contributing to *E*_p_.

The combined data for 1H, 2H (and 1^+^, 2^+^) and the deviation of 1^+^ from the *E*_p_*vs.* Δ*G*_H–_ correlation lines are consistent with differences in reorganization energy that can be rationalized by the structural changes between the 1^+^ and 1H pair. We expect no large structural changes upon conversion to 1H and so it is reasonable to expect that kinetic contributions to *E*_p_ might be relatively low. In contrast, there are obvious structural differences between the 2^+^/2H pair. For 2^+^ we know that the flanking pyrazolyl rings of pz_2_P have *N*-donor atoms oriented in toward the cationic *N*-Me-pyridyl group, and in 2H those *N*-donor atoms of the pyrazolyl rings rotate away since the py ring is no longer cationic, as in the known structure of pz_2_P where the pyrazolyl N atoms rotate out.^36^ An additional effect of the Group 13 cation may be to stabilize the DHP^−^*via* bonding interactions between the N_py_ π-electrons and *p*_z_ orbital of the metal: electron donation from N_py_ to group 13 3+ cations have been observed in other complexes of tridentate pyridyl-centered ligands,^49^ and may stabilize the DHP^−^.^50^

## Conclusion

New compounds 1H and 2H were prepared by a direct reaction of a hydride donor with the neutral ligand complexes 1^+^, and 2^+^, respectively: this hydride transfer reaction is a formal two-electron reduction and single protonation. Measurements of the *E*_p_ and Δ*G*_H–_ for 1^+^, 2^+^, and 4^+^, show that 1^+^ and 4^+^ do not follow the *E*_p_*vs.* Δ*G*_H–_ scaling relationship. Both 1^+^ and 4^+^ have *N*-pyridyl coordination to Group 13 3+ cations, whereas 2^+^ is *N*-alkylated. These data were collected in THF and MeCN where possible, and the work in MeCN additionally permitted some quantification of this effect, where 2^+^ is shifted anodically from the *E*_p_*vs.* Δ*G*_H–_ relationship by 215 mV (Fig. 2). Some of the observed anodic shift may arise from the cationic nature of [(pz_2_P)E]^+^ but it is unlikely that the full 215 mV anodic shift can be attributed to a single positive charge given that all of the model organohydrides on the plot have equivalent cationic charge. Based on these results, we propose that *N*-coordination of a Group 13 3+ cation to pyridyl offers a strategy for kinetic lowering of *E*_p_. Future work on well-designed *N*-metallated DHP^−^s will target electrochemically-driven HT reaction chemistry.

## Author contributions

Leo Parsons collected and analyzed the data, and was involved in writing the manuscript. James Fettinger was involved in refinement of solid state structural X-ray data. Louise Berben was responsible for project design and management, and writing the manuscript.

## Conflicts of interest

There are no conflicts to declare.

## Supplementary Material

SC-014-D3SC03806H-s001

SC-014-D3SC03806H-s002
