# Supplementary material for: Group 13 ion coordination to pyridyl breaks the reduction potential vs. hydricity scaling relationship for dihydropyridinates
Source: Chem Sci. 2023 Nov 16;14(47):13944–50. doi: 10.1039/d3sc03806h (PMC10699567; doi:10.1039/d3sc03806h)
Supplement: SC-014-D3SC03806H-s001 [file SC-014-D3SC03806H-s001.pdf]

Electronic Supplementary Material (ESI) for Chemical Science.
This journal is © The Royal Society of Chemistry 2023

Supporting Information

Group 13 ion coordination to pyridyl breaks the reduction potential vs hydricity scaling relationship for dihydropyridinates.

Leo W. T. Parsons, James C. Fettinger, and Louise A. Berben*

Department of Chemistry, University of California, Davis CA 95616

e-mail: laberben@ucdavis.edu

Table of Contents

1. Experimental Methods

2. Calculations

Calculation S1: General determination of species concentration during equilibrium reactions monitored by ^{1}H NMR.

Calculation S2: Determination of equilibrium constants for equilibrium reactions between **1**-GaCl4 with **2**H and **4**-$AlCl_4$ with **2**H.

Calculation S3: Hydricity difference between species in equilibrium.

3. Tables

Table S1: Crystallographic data for compounds **1**H and **3**H.

Table S2: Selected bond lengths, angles and τ_5 values for **1**H and **3**H.

Table S3: Selected pyridine bond metrics for **4**H, **1**H and **3**H.

Table S4: Selected bond angles and torsions for **4**H, **1**H, and **3**H

4. Figures

Figure S1: ^{1}H NMR and ^{13}C NMR of **3**H.

Figure S2: ^{1}H NMR and ^{13}C NMR of **1**H.

Figure S3: ^{1}H NMR and ^{13}C NMR of **2**H.

Figure S4: Time dependent ^{1}H NMR spectra of reaction between **2-**OTf and 1,3-simethyl-2-phenyl-2,3-dihydro-1*H*-benzo[d]imidazole.

Figure S5: Time dependent ^{1}H NMR spectra of reaction between **2**H and 1,3-dimethyl-1*H*-benzimidazolium iodide.

Figure S6: Time dependent ^{1}H NMR spectra of **2**$^{+}$-OTf and concentration vs time plot.

Figure S7: Time dependent ^{1}H NMR spectra of **2**H and concentration vs time plot.

Figure S8: Time dependent ^{1}H NMR spectra of 1,3-dimethyl-2-phenyl-2,3-dihydro-1H-benzo[d]imidazole and concentration vs time plot.

Figure S9: Time dependent ^{1}H NMR spectra of 1,3-dimethyl-1*H*-benzimidazolium iodide and concentration vs time plot.

Figure S10: Reaction scheme and time-dependent ^{1}H NMR spectra for reaction of **1**-$GaCl_4$ with **2**H.

Figure S11: Reaction scheme and time-dependent ^{1}H NMR spectra for reaction of **4**-$AlCl_4$ with **2**H.

Figure S12: Concentration (mM) and mol % vs time (hours) for reaction of **1**-$GaCl_4$ with **2**H and reaction of **4**-$AlCl_4$ with **2**H.

5. References

Experimental Section

Preparation of Compounds. All manipulations were carried out using standard Schlenk or glovebox techniques under a dinitrogen atmosphere. Unless otherwise noted, solvents were deoxygenated and dried by thorough sparging with Ar gas followed by passage through an activated alumina column. Deuterated solvents were purchased from Cambridge Isotopes Laboratories, Inc. and were degassed and stored over activated 3 Å molecular sieves prior to use. 2,6-bis(5-isobutyl-1-isopropyl-1H-pyrazol-3-yl)pyridine (pz_2P),[1] **4**-$AlCl_4$,[1] and 1,3-dimethyl-1*H*-benzimidazolium iodide,[2] were synthesized according to literature methods. All other reagents were purchased from commercial vendors and used without further purification.

($pz_2^{H}P^{-}$)$GaCl_2$ (1H). 25 mg (0.059 mmol) compound **2**H was dissolved in 500 µL dry CD_3CN and transferred to a J-Young tube in a dry N_2 filled glovebox. 49.3 mg (0.065 mmol) of **1**-$GaCl_4$ was dissolved in 500 µL CD_3CN and transferred to the J-young tube with compound **2**H. After letting the reaction sit undisturbed for 3 days, purple crystals of **1**H grew around the walls of the J-Young tube which were suitable for X-ray diffraction. The CD_3CN solution was decanted, and the crystals were washed with Et_2O brought up in benzene and lyophilized to give 14.9 mg (56% yield) of **1**H as a pink powder. ^1H NMR (600 MHz, C_6D_6): δ 5.92 (s, 2H), 5.50 (hept., *J* = 7.2 Hz, 2H), 4.94 (t, *J* = 3.5 Hz, 2H), 3.48 (t, *J* = 3.5 Hz, 2H), 2.07 (d, *J* = 7.2 Hz, 4H), 1.53 (m, 2H), 1.34 (d, *J* = 7.2 Hz, 12H), 0.65 (d, *J* = 6.6 Hz, 12H). ^{13}C NMR (151 MHz, C_6D_6): δ 146.38, 146.02, 133.91, 100.15, 94.70, 54.96, 35.39, 28.17, 25.14, 22.45, 21.77. Anal Calcd for $C_{25}H_{38}Cl_2N_5Ga$: C, 54.67; H, 6.97; N, 12.75 Found: C, 54.39; H, 6.69; N, 12.36.

($pz_2^{H}P$)CH_3 (2H). To a solution of 50 mg (0.088 mmol) **2**-OTf in anhydrous THF added 114 µL (0.114 mmol) of a 1 M solution of $LiEt_3BH$ in THF. The solution turned from colorless to deep yellow quickly following the addition of the $LiEt_3BH$. The reaction solution was concentrated to dryness after 5 minutes. The concentrate was dissolved in benzene, filtered over celite and the solvent was removed *in vacuo*. The resulting oil was dissolved in hexanes, filtered and dried *in vacuo* to yield **2**H as a yellow oil (10 mg, 27 % yield). ^1H NMR (600 MHz, C_6D_6): δ 6.20 (s, 2H), 5.62 (m, 2H), 3.98 (hept, *J* = 6.7 Hz, 2H), 3.27 (m, 3H), 3.24 (m, 2H), 2.15 (d, *J* = 7.3 Hz, 4H), 1.65 (hept, *J* = 7.0 Hz, 2H), 1.34 (d, *J* = 6.5 Hz, 12H), 0.80 (d, *J* = 6.5 Hz, 12H). ^{13}C NMR (151 MHz, C_6D_6): δ 148.47, 140.16, 140.06, 103.84, 48.96z, 38.52, 34.21, 31.56, 28.24, 24.31, 22.59, 22.14. (ESI-HRMS) m/z: $[M+H]^+$. Calcd. for $C_{26}H_{41}N_5$ 424.3435, Found 424.3420.

($pz_2^{iPr}HP^{-}$)$GaCl_2$ (3H). A solution of 500 mg (1.23 mmol) pz_2P in THF was added 56.5 mg (2.43 mmol) of sodium and stirred until all the sodium was consumed, producing a deep red solution. 216 mg (1.23 mmol) of $GaCl_3$ in THF was then added dropwise and stirred an additional two hours during which time a brown/purple solution was produced. THF was removed *in vacuo* and the resulting powder dissolved in diethyl ether, filtered over celite, and concentrated (156 mg, 22 %). Crystals suitable for X-ray diffraction were grown as colorless plates from a saturated hexane solution. ^1H NMR (600 MHz, C_6D_6): δ 5.97 (s, 2H), 5.48 (hept, *J* = 7.3 Hz, 2H), 5.06 (d, *J* = 3.8 Hz, 2H), 3.52 (q, *J* = 4.0 Hz, 1H), 2.08 (d, *J* = 7.2 Hz, 4H), 1.76 (m, 1H), 1.53 (mr, 2H), 1.32 (m, 12H), 1.12 (d, *J* = 6.9 Hz, 6H), 0.64 (m, 12H). ^{13}C NMR (151 MHz, C_6D_6) δ: 146.45, 146.14, 122.96, 100.27, 97.36, 54.97, 42.03, 36.74, 35.42, 28.20, 22.51, 21.79, 21.76, 19.30. Anal Calcd for $C_{28}H_{44}Cl_2N_5Ga$: C, 56.87; H, 7.5; N, 11.84 Found: C, 56.66; H, 7.48; N, 11.95.

Reactions to determine equilibria. Hydride transfer equilibria between **4**-$AlCl_4$ or **1**-GaCl4 with **2**H were monitored by ^1H NMR spectroscopy in C_6D_6. Hydride transfer equilibria between **2**H and 1,3-dimethyl-2-phenyl-2,3-dihydro-1*H*-benzo[d]imidazole as well as $\mathbf{2}^+$-OTf and 1,3-dimethyl-2-phenyl-2,3-

dihydro-1*H*-benzo[d]imidazole were monitored by ^{1}H NMR spectroscopy in CD_3CN. Diagnostic signals for each species in solution were referenced to an internal standard of trimethoxy benzene. Sample preparation for each experiment is outlined below.

Reaction of 2H with 1-GaCl$_4$. Reaction solution was prepared by weighing 5 mg (0.012 mmol, 1 eq.) of **2**H in a 20 mL vial and dissolving in 400 µL C_6D_6 and transferring to a NMR tube. To a separate vial was weighed first 8.9 mg (0.012 mmol, 1 eq.) **1**-$GaCl_4$, then 7.3 mg (0.040 mmol, 3.3 eq.) trimethoxy benzene was added to vial. The solid **1**-$GaCl_4$ and trimethoxy benzene were brought up in 400 µL C_6D_6 and transferred to NMR tube with **2**H. The vial containing residual **1**-$GaCl_4$ and trimethoxy benzene was then washed with an additional 100 µL C_6D_6 and transferred to the NMR tube.

Reaction of 2H with 4-AlCl$_4$. Reaction solution was prepared by weighing 5 mg (0.01 mmol, 1 eq.) of **2**H in a 20 mL vial and dissolving in 400 µL C_6D_6 and transferring to a NMR tube. To a separate vial was weighed first 7.9 mg (0.01 mmol, 1 eq.) **4**-$AlCl_4$, then 2.0 mg (0.01 mmol, 1 eq.) trimethoxy benzene was added to vial. The solid **4**-$AlCl_4$ and trimethoxy benzene were brought up in 300 µL C_6D_6 and transferred to the NMR tube with **2**H.

Reaction of 2-OTf and 1,3-dimethyl-2-phenyl-2,3-dihydro-1*H*-benzo[d]imidazole. Reaction solution was prepared by weighing 13 mg (0.02 mmol, 1 eq.) of **2**-OTf in a 20 mL vial and dissolving in 500 µL CD_3CN and transferring to a NMR tube. To a separate vial was weighed first 5.4 mg (0.02 mmol, 1 eq.) 1,3-dimethyl-2-phenyl-2,3-dihydro-1H-benzo[d]imidazole, then 4.0 mg (0.02 mmol, 1 eq.) trimethoxy benzene was added to vial. The solid 1,3-dimethyl-2-phenyl-2,3-dihydro-1*H*-benzo[d]imidazole and trimethoxy benzene were brought up in 500 µL CD_3CN and transferred to the NMR tube with **2**-OTf.

Reaction of 2H and 1,3-dimethyl-1*H*-benzimidazolium iodide. Reaction solution was prepared by weighing 10 mg (0.024 mmol, 1 eq.) of **2**H in a 20 mL vial and dissolving in 500 µL CD_3CN and transferring to a NMR tube. To a separate vial was weighed first 6.6 mg (0.024 mmol, 1 eq.) 1,3-dimethyl-1*H*-benzimidazolium iodide, then 4.0 mg (0.024 mmol, 1 eq.) trimethoxy benzene was added to vial. The solid 1,3-dimethyl-1*H*-benzimidazolium iodide and trimethoxy benzene were brought up in 500 µL CD_3CN and transferred to the NMR tube with **2**H.

X-ray Structure Determinations. X-ray diffraction studies were carried out on a Bruker SMART APEX Duo diffractometer equipped with a CCD detector.[3] Measurements were carried out at −175 °C using Mo Kα (0.71073 Å) and Cu Kα (1.54178 Å) radiation. Crystals were mounted on a Kaptan loop with paratone-N oil. The initial lattice parameters were obtained from least-squares analysis of more than 100 centered reflections; these parameters were later refined against all data. Data were integrated and corrected for Lorentz polarization effects using SAINT[4] and were corrected for absorption effects using SADABS2.3.[5] Space-group assignments were based on systematic absences, E statistics, and the successful refinement of the structures. Structures were solved by direct methods with the aid of successive difference Fourier maps and were refined against all data using the SHELXTL 2014/7 software package.[6] Thermal parameters for all non-H atoms were refined anisotropically. H atoms, where added, were assigned to ideal positions, and refined using a riding model with an isotropic thermal parameter 1.2 times that of the attached C atom (1.5 times for methyl H atoms).

Other Physical Measurements. ^{1}H and ^{13}C NMR spectra were recorded at ambient temperature using a Varian 600 MHz spectrometer. Chemical shifts were referenced to a residual solvent. Elemental analyses were performed by the Microanalytical Laboratory at The University of California, Berkeley. For

HRMS analysis, samples were analyzed by flow-injection analysis into a Thermo Fisher Scientific LTQ Orbitrap XL (San Jose, CA) operated in the centroided mode. Samples were injected into a mixture of 50% MeOH and 0.1% Formic Acid/H20 at a flow of 200 ul/min. Source parameters were 5kV spray voltage, capillary temperature of 275̊C and sheath gas setting of 20. Spectral data were acquired at a resolution setting of 100,000 FWHM with the lockmass feature which typically results in a mass accuracy < 2 ppm.

2. Calculations and Equations

Calculation S1. Determination of species concentration during equilibrium reactions monitored by ^{1}H NMR.

Diagnostic signals for all peaks were integrated over a constant ppm range for each ^{1}H NMR spectrum recorded. Integration of the O-C$\underline{H_3}$ signal from the trimethoxy benzene standard was set to 100.00 for each spectrum and concentrations of diagnostic signals were calculated from the peak integration relative to internal standard. Using equation S1

$$[X] = \frac{I_X * (9/H_X)}{100} * [Std.] \qquad \text{(equation S1)}$$

where [X] is the concentration of the species of interest, I_X is the raw integration of the species of interest, H_X is the number of protons corresponding to the signal of interest and [Std.] is the concentration of internal standard.

Calculation S2.

Equilibrium constants were calculated based off the concentration of each species at equilibrium determined by ^{1}H NMR (Figure 2).

$$K = \frac{[1 - GaCl_4][2H]}{[1H][2^+]} \qquad \text{(equation S2)}$$

Equilibrium concentrations were taken from empirical data at 94 hours for the reaction of **1**-$GaCl_4$ and **2**H (K_1) and at 94 hours for reaction of **4**-$AlCl_4$ and **2**H (K_2).

Equilibrium constant for the reaction of **1**-$GaCl_4$ with **2**H (K_1).

$$K_1 = \frac{[1 - GaCl_4][2H]}{[1H][2]} = \frac{4.00 * 3.13}{1.86 * 8.13} = 0.83$$

Equilibrium constant for the reaction of **4-**$AlCl_4$ with **2**H (K_2).

$$K_2 = \frac{[4 - AlCl4][2H]}{[4H][2]} = \frac{9.328 * 0.912}{4.401 * 8.119} = 0.24$$

Calculation S3. Hydricity difference between two species in equilibrium.

$$\Delta G_{H^-} = -RTln(K) \qquad \text{(Equation S3)}$$

Calculated difference in hydricity between **2**H and **1**-$GaCl_4$.

$$\Delta G_{H-}1 = +\ 0.1 \pm 0.1\ (\text{kcal mol}^{-1})$$

Calculated difference in hydricity between **2**H and **4**-$AlCl_4$.

$$\Delta G_{H-}2 = +\ 0.8 \pm 0.1\ (\text{kcal mol}^{-1})$$

3. Tables

Table S1. Crystallographic data for compounds **1**H and **3**H.

	**1**H [a]	**3**H [b]
Formula	$C_{25}H_{38}Cl_2GaN_5$	$C_{28}H_{44}Cl_2GaN_5$
Crystal size	0.612 × 0.251 × 0.134	0.385 × 0.174 × 0.066
Formula weight, g mol^{-1}	549.22	591.30
Space group	$P2_1/c$	$P2_1$/n
a, Å	13.1613(11)	12.1606(3)
b, Å	13.2286(11)	15.1860(4)
c, Å	15.7223(13)	16.3042(4)
α, deg	90	90
β, deg	93.540(2)	90.4970(13)
γ, deg	90	90
V, Å^3	2732.1(4)	3010.80(13)
Z	4	4
T, K	100(2)	100(2)
ρ, calcd, g cm^{-3}	1.335	1.304
Refl. Collected / $2\theta_{max}$	32223 / 61.46	16451 / 144.74
Unique refl. / $I > 2\sigma(I)$	8344 / 7871	5910 / 5063
No. Parameters / restraints	450 / 0	375 / 57
λ,Å/μKα,cm^{-1}	0.71073	1.54178
R_1/GOF	0.0203 / 1.057	0.0399 / 1.038
$wR_2(I > 2\sigma(I))^b$	0.0530	0.1048
Residual density, e Å^{-3}	0.421 / -0.249	0.877 / -0.437

[a] Obtained with graphite-monochromated Mo Kα (λ = 0.71073 Å) radiation. [b] Obtained with graphite-monochromated Cu Kα (λ = 1.54178 Å) radiation [c] $R1 = \Sigma|| F_o | - F_c||/\Sigma|F_o|$, $wR2 = \{\Sigma[w(F_o^2-F_c^2)^2]/\Sigma[w(F_o^2)^2]\}^{1/2}$.

Table S2. Selected bond length and angles for **1**H and **3**H.

	**1**H	**3**H
N_{pz}-C_{pz}	1.340(1)	1.344(3)
C_{pz}-C_o	1.463(1)	1.451(9)
N_{py}-C_o	1.386(1)	1.405(9)
C_o-C_m	1.344(1)	1.37(1)
C_m-C_{py}	1.508(1)	1.505(8)
C_{py}-$C_{m'}$	1.511(1)	1.509(7)
$C_{m'}$-$C_{o'}$	1.346(1)	1.29(2)
N_{py}-$C_{o'}$	1.391(1)	1.46(2)
$C_{o'}$-$C_{pz'}$	1.464(1)	1.46(2)
$N_{pz'}$-$C_{pz'}$	1.340(1)	1.339(3)
N_{py}-X	1.8991(9)	1.899(2)
τ_5 [7]	0.76	0.76

Table S3. Selected pyridine bond metrics for **4**H,[1] **1**H, and **3**H. See Figure 1 for atom labelling Scheme.

	N_{py}-C_o	C_0-C_m	C_m-C_p	C_p-$C_{m'}$	$C_{m'}$-$C_{o'}$	$C_{o'}$-N_{py}	N_{py}-E
**4**H	1.3894(19)	1.333(2)	1.509(2)	1.511(2)	1.338(2)	1.3922(19)	1.869(1)
**1**H	1.389(1)	1.345(2)	1.511(2)	1.504(2)	1.342(2)	1.386(1)	1.898(1)
**3**H	1.37(1)	1.29(2)	1.507(7)	1.510(8)	1.38(1)	1.340(9)	1.900(2)

Table S4: Selected bond angles and torsions for, **4**H,[1] **1**H, and **3**H. See Figure 1 for atom labelling Scheme.

	Torsion angle N_{pz}-C_{pz}-C_o-N_{py}	Torsion angle′ $N_{pz'}$ -$C_{pz'}$-$C_{o'}$-N_{py}	N_{pz}-M-$N_{pz'}$ angle	Torsion angle M-N_{py}-C_o-C_{pz}	Torsion angle M-N_{py}-$C_{o'}$-$C_{pz'}$	Average of M-N_{py}-C_o-C_{pz} and M-N_{py}-$C_{o,}$-$C_{pz'}$	N_{pz}-C_{pz}-$N_{pz'}$-$C_{pz'}$ torsion
**4**H	3.18	4.50	156.71(5)	4.60	1.82	3.21	14.25
**1**H	3.88	5.04	156.47(3)	5.18	1.77	3.48	15.75
**3**H	1.58	12.22	156.51(8)	7.44	8.95	9.2	14.77

4. Figures

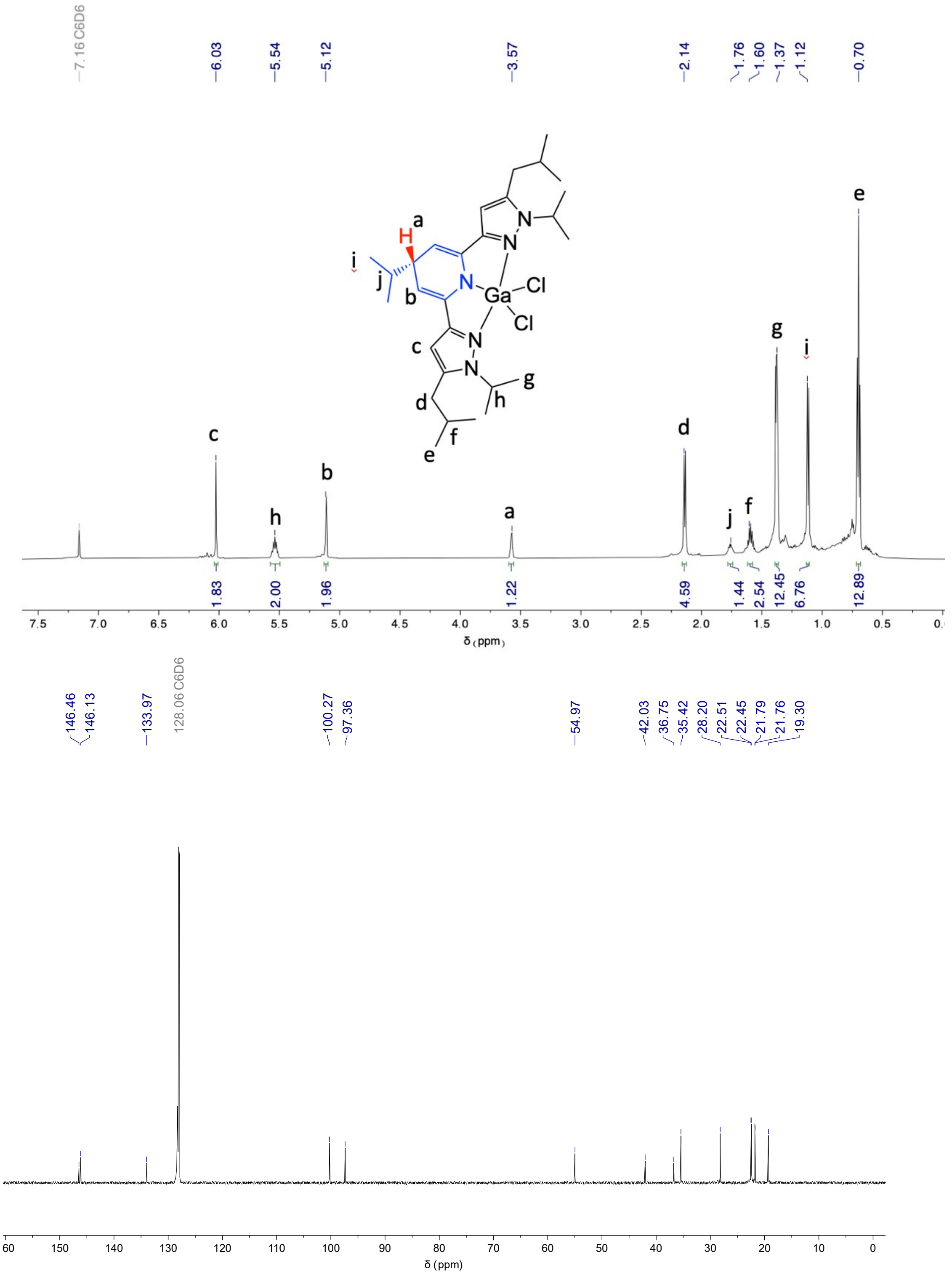

Figure S1. ^{1}H NMR (top) and ^{13}C NMR (bottom) spectra of **3**H (C_6D_6).

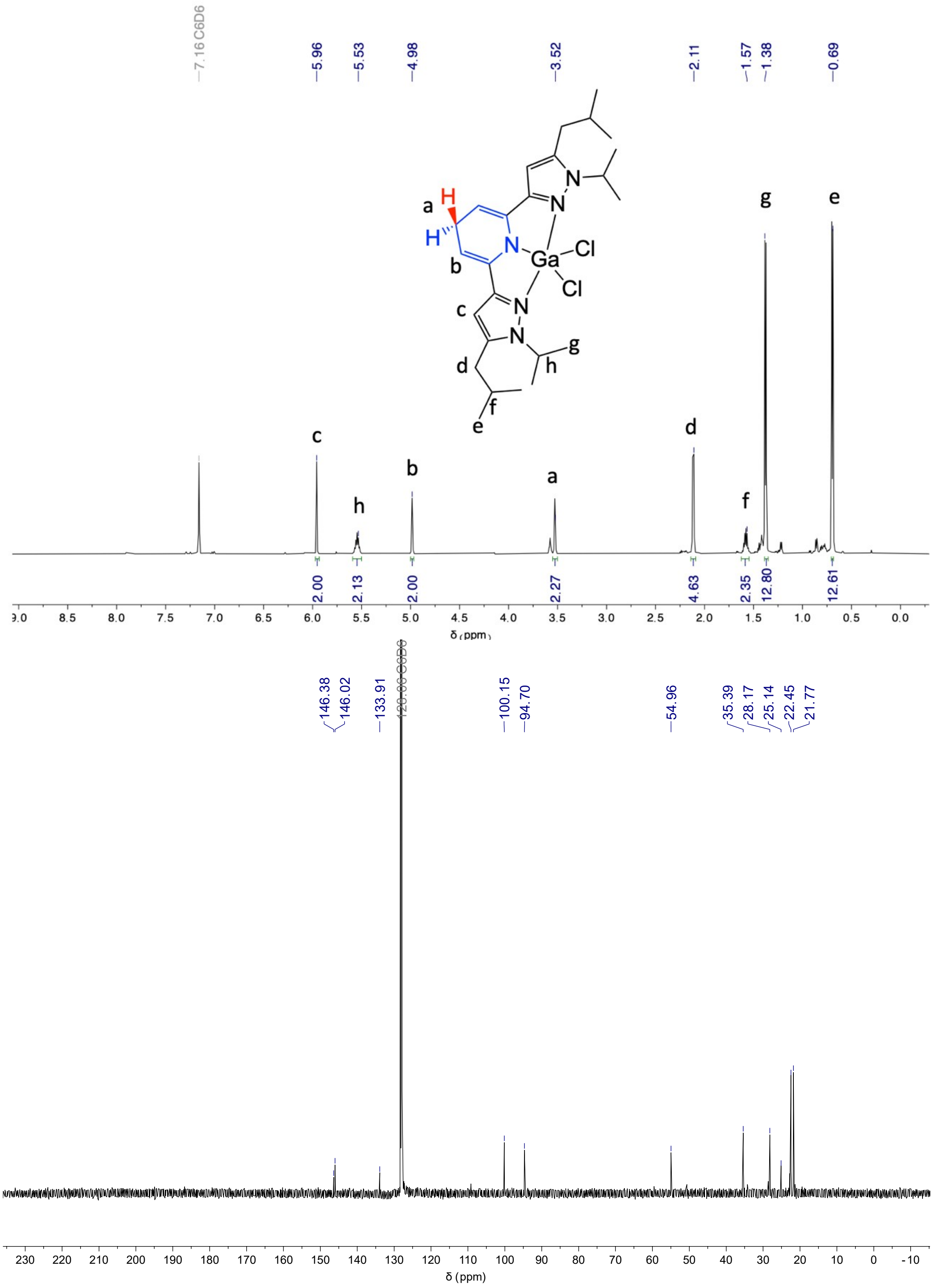

Figure S2. ^{1}H NMR (top) and ^{13}C NMR (bottom) spectra of **1**H (C_6D_6).

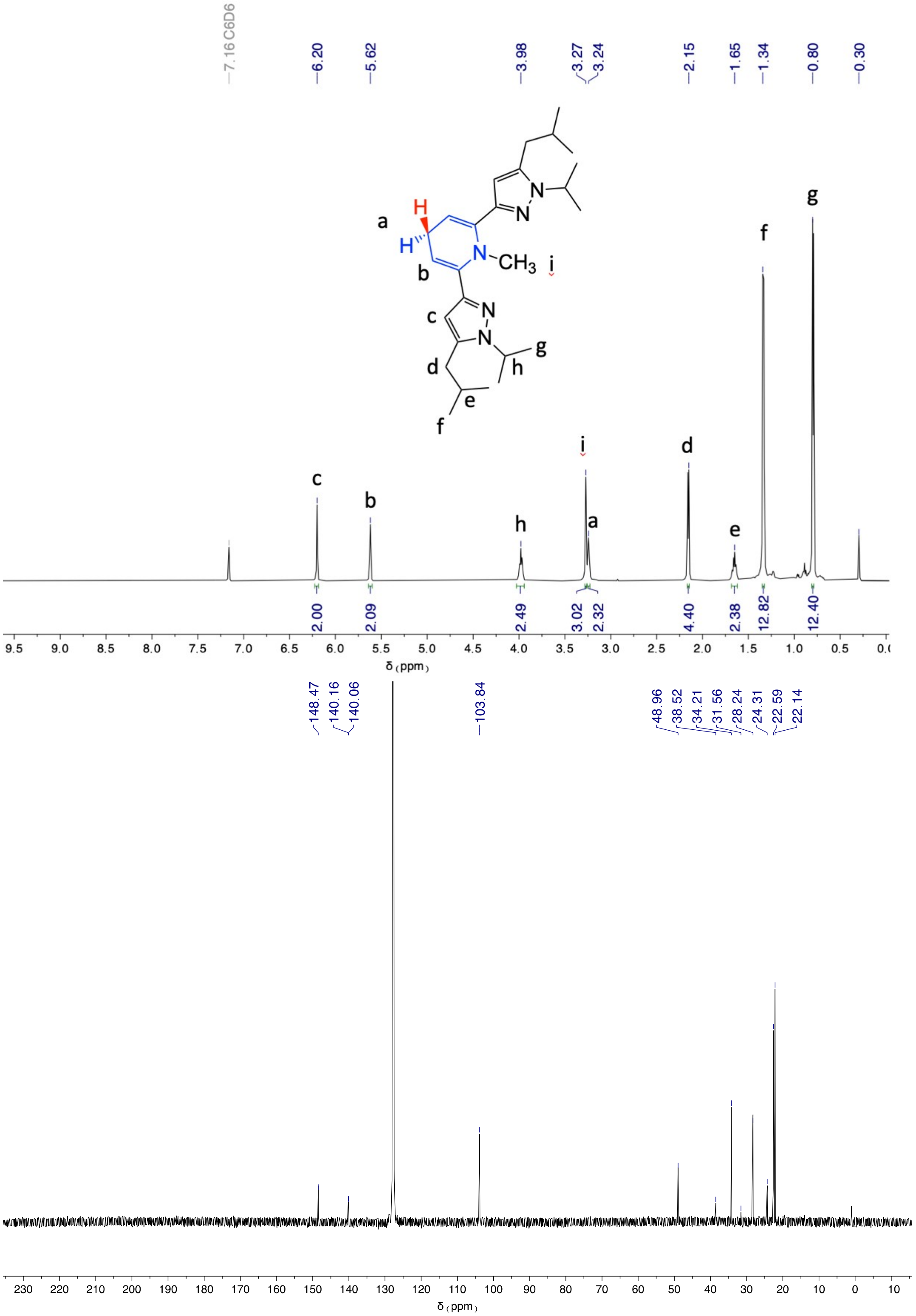

Figure S3. ^{1}H NMR (top) and ^{13}C NMR (bottom) spectra of **2**H (C_6D_6).

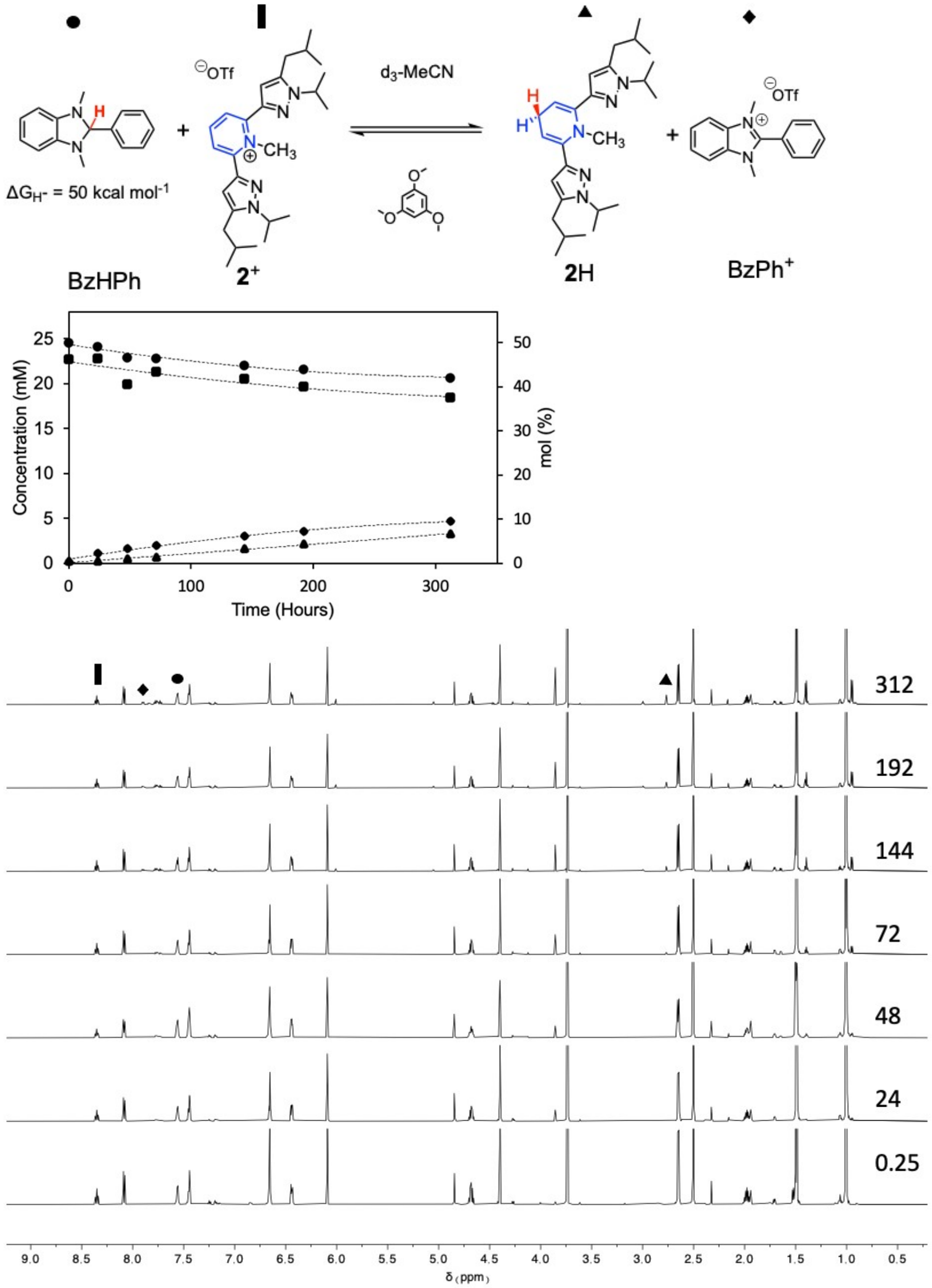

Figure S4: Reaction scheme (top), time dependent ^{1}H NMR, concentration vs time plots (middle) and spectra of reaction between **2-**OTf and 1,3-dimethyl-2-phenyl-2,3-dihydro-1*H*-benzo[d]imidazole at 0.25, 24, 48, 72, 144, 192 and 312 hours in CD_3CN with trimethoxy benzene as an internal standard (bottom). Circles, squares, triangles and diamonds correspond to compounds and their diagnostic ^{1}H NMR signal for 1,3-dimethyl-2-phenyl-2,3-dihydro-1*H*-benzo[d]imidazole (BzHPh), compound **2**$^{+}$, **2**H and 1,3-dimethyl-2-phenyl-1*H*-benzo[d]imidazole-3-ium triflate (BzPh) respectively.

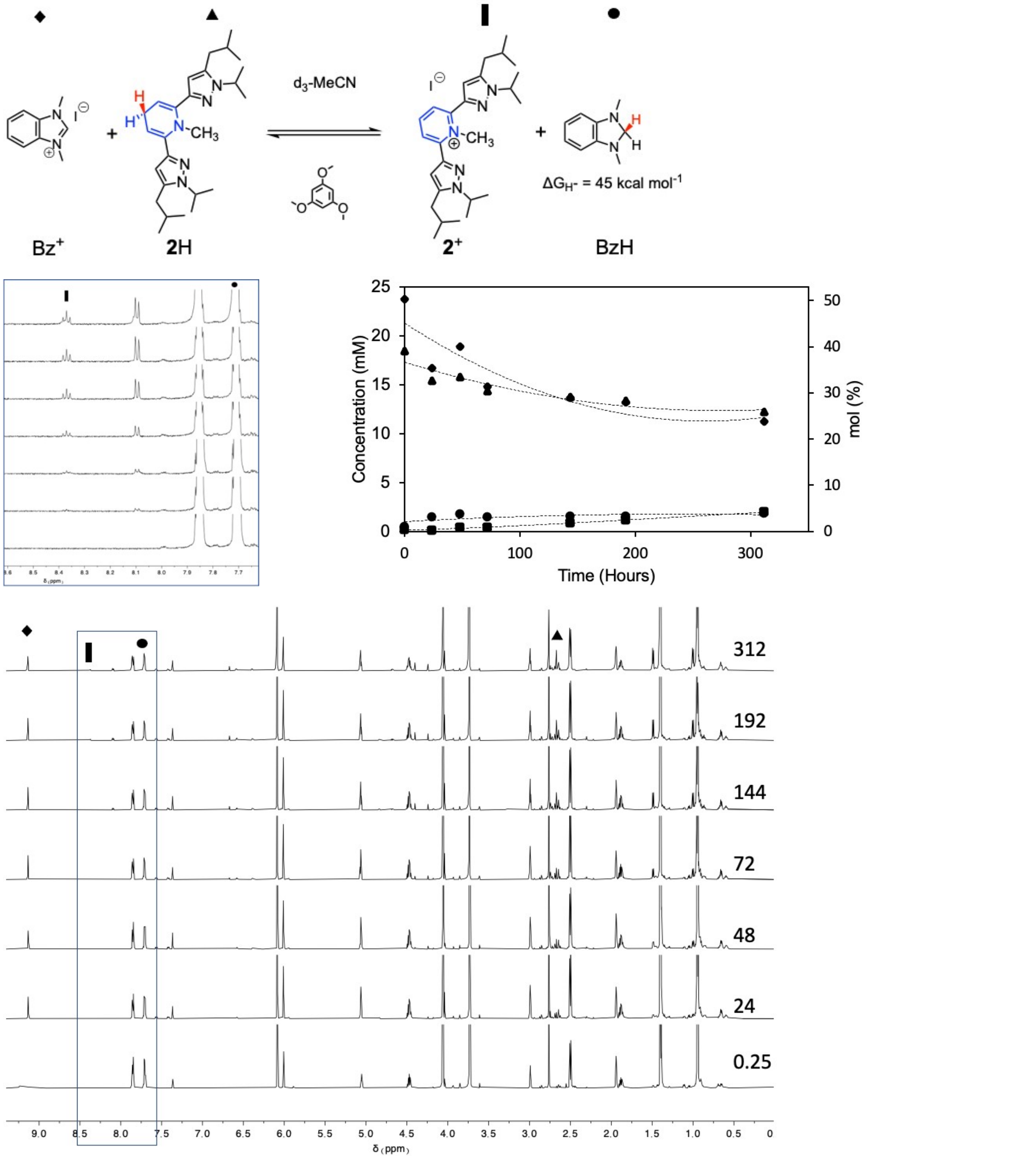

Figure S5: Reaction scheme (top), concentration vs time plots and amplified ^{1}H NMR spectra (middle) and time dependent ^{1}H NMR Spectra of reaction between **2**H and 1,3-dimethyl-1*H*-benzimidazolium iodide at 0.25, 24, 48, 72, 144, 192 and 312 hours in CD_3CN with trimethoxy benzene as an internal standard (bottom). Blue boxed area corresponds to region highlighted in the middle of the bottom spectra. Diamonds, triangles, squares, and circles correspond to compounds and their diagnostic ^{1}H NMR signal for 1,3-dimethyl-1*H*-benzimidazolium iodide (Bz^{+}), compound **2**H, $\mathbf{2}^{+}$ and 1,3-dimethyl-2,3-dihydro-1*H*-benzo[d]imidazole (BzH) respectively.

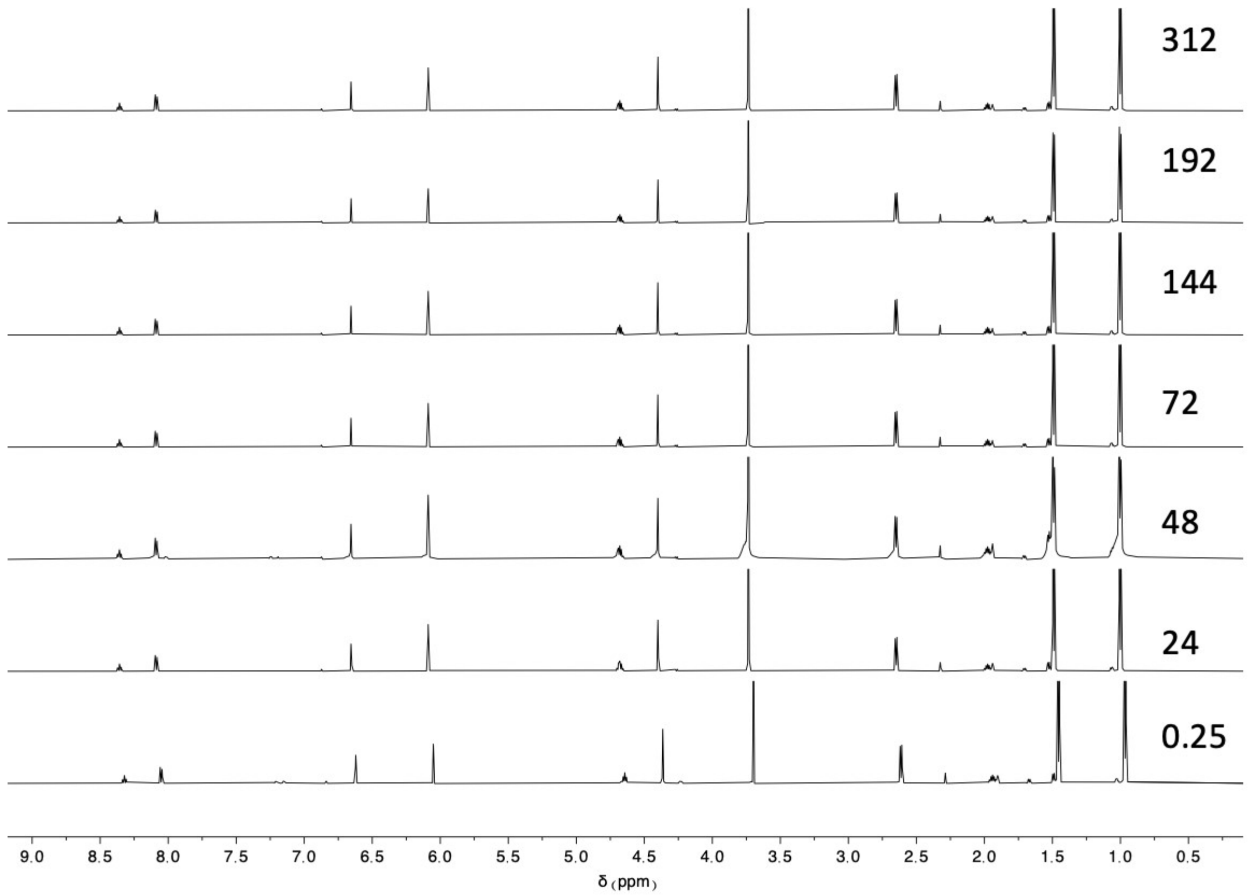

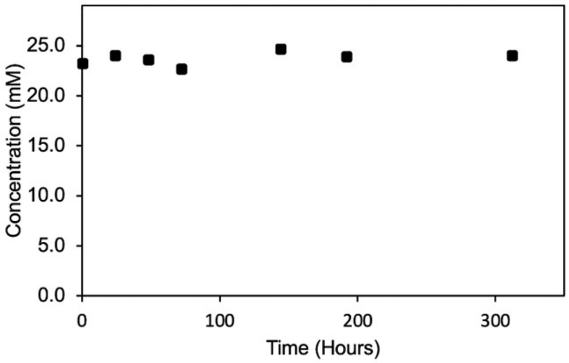

Figure S6: Time dependent ^{1}H NMR spectra of **2**$^{+}$ in CD_3CN with trimethoxy benzene as an internal standard recorded at 0.25, 24, 48, 72, 144, 192 and 312 hours (top) and corresponding concentration vs time plot (bottom).

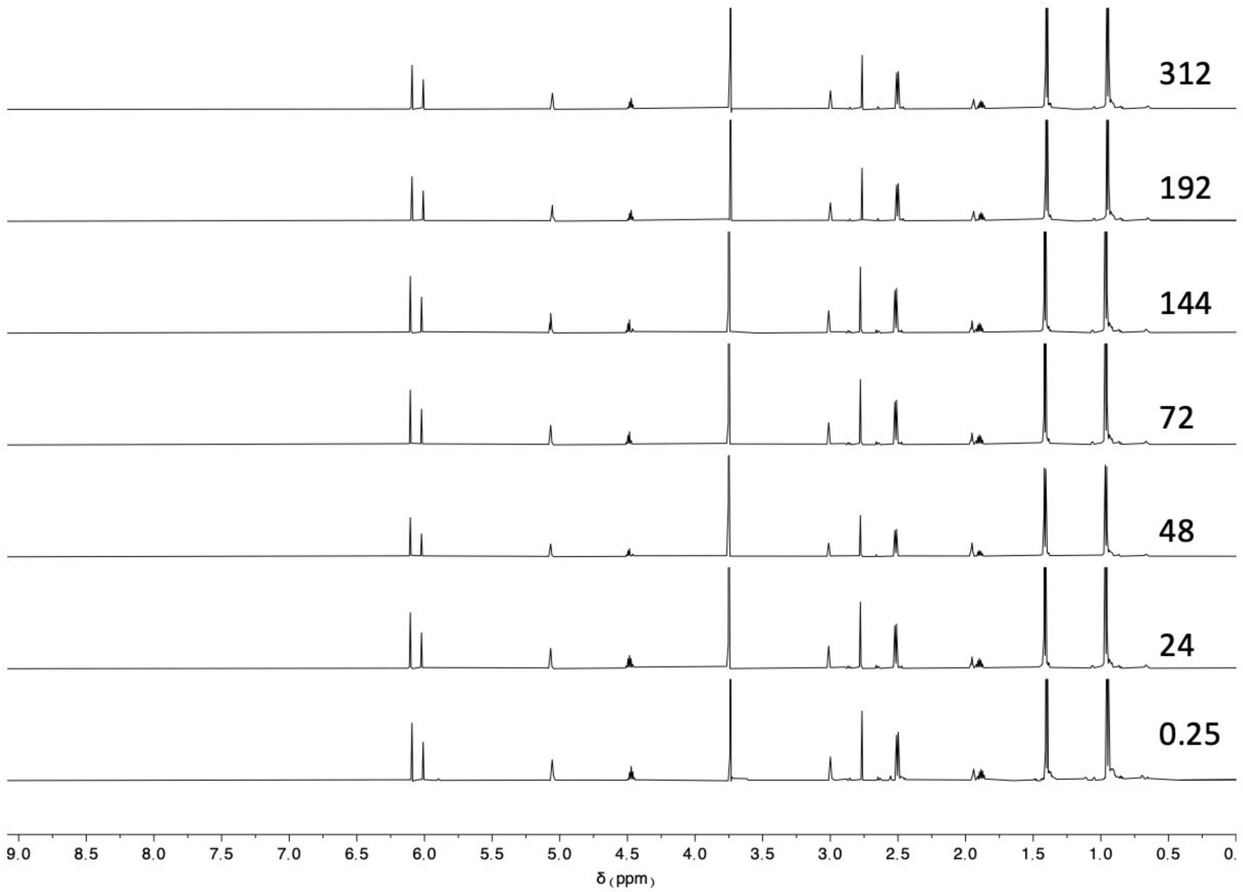

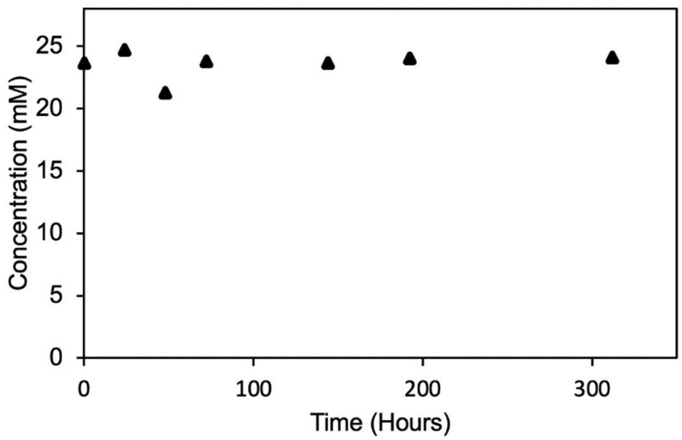

Figure S7: Time dependent ^{1}H NMR spectra of **2**H in CD_3CN with trimethoxy benzene as an internal standard recorded at 0.25, 24, 48, 72, 144, 192 and 312 hours (top) and corresponding concentration vs time plot (bottom).

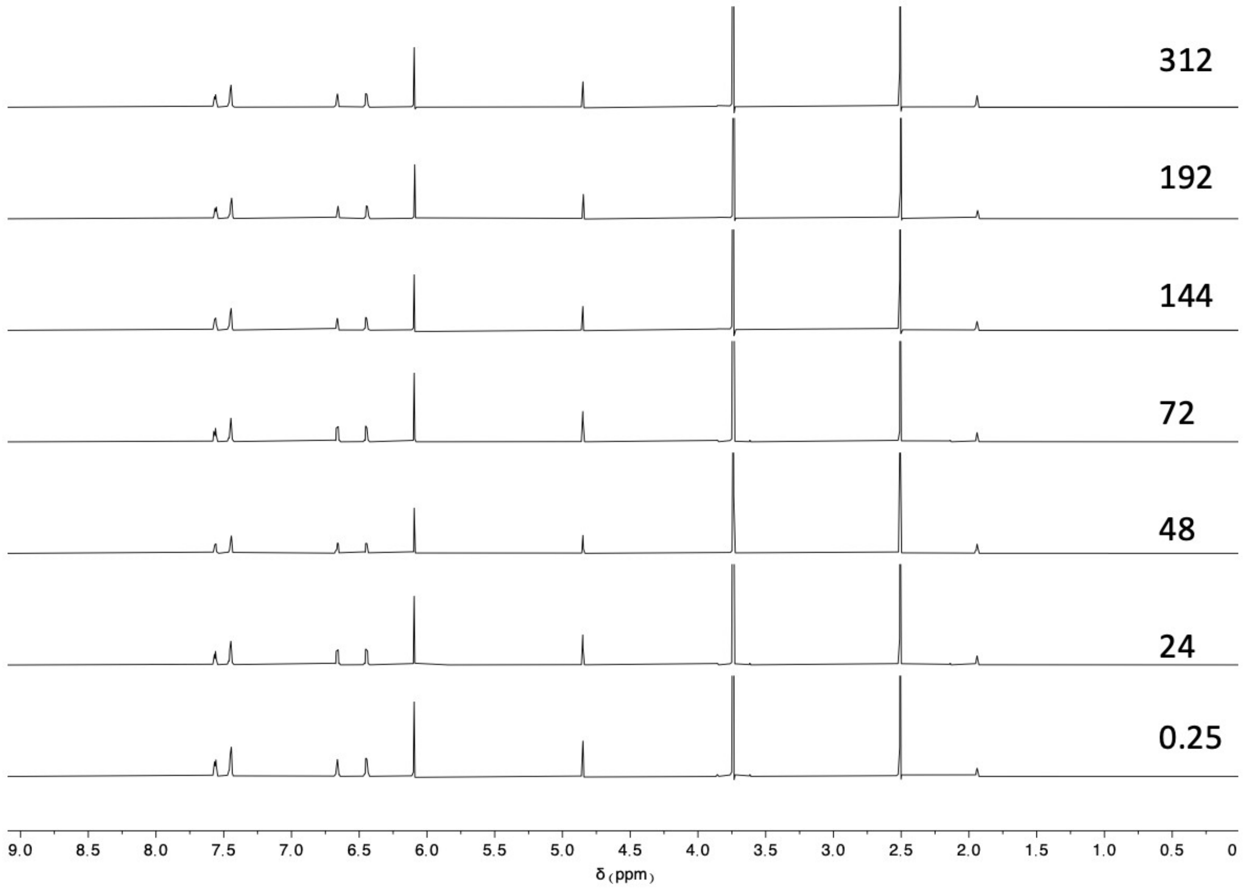

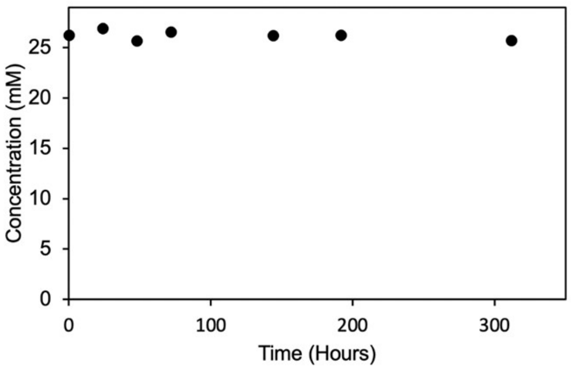

Figure S8: Time dependent ^{1}H NMR spectra of 1,3-dimethyl-2-phenyl-2,3-dihydro-1*H*-benzo[d]imidazole in CD_3CN with trimethoxy benzene as an internal standard recorded at 0.25, 24, 48, 72, 144, 192 and 312 hours (top) and corresponding concentration vs time plot (bottom).

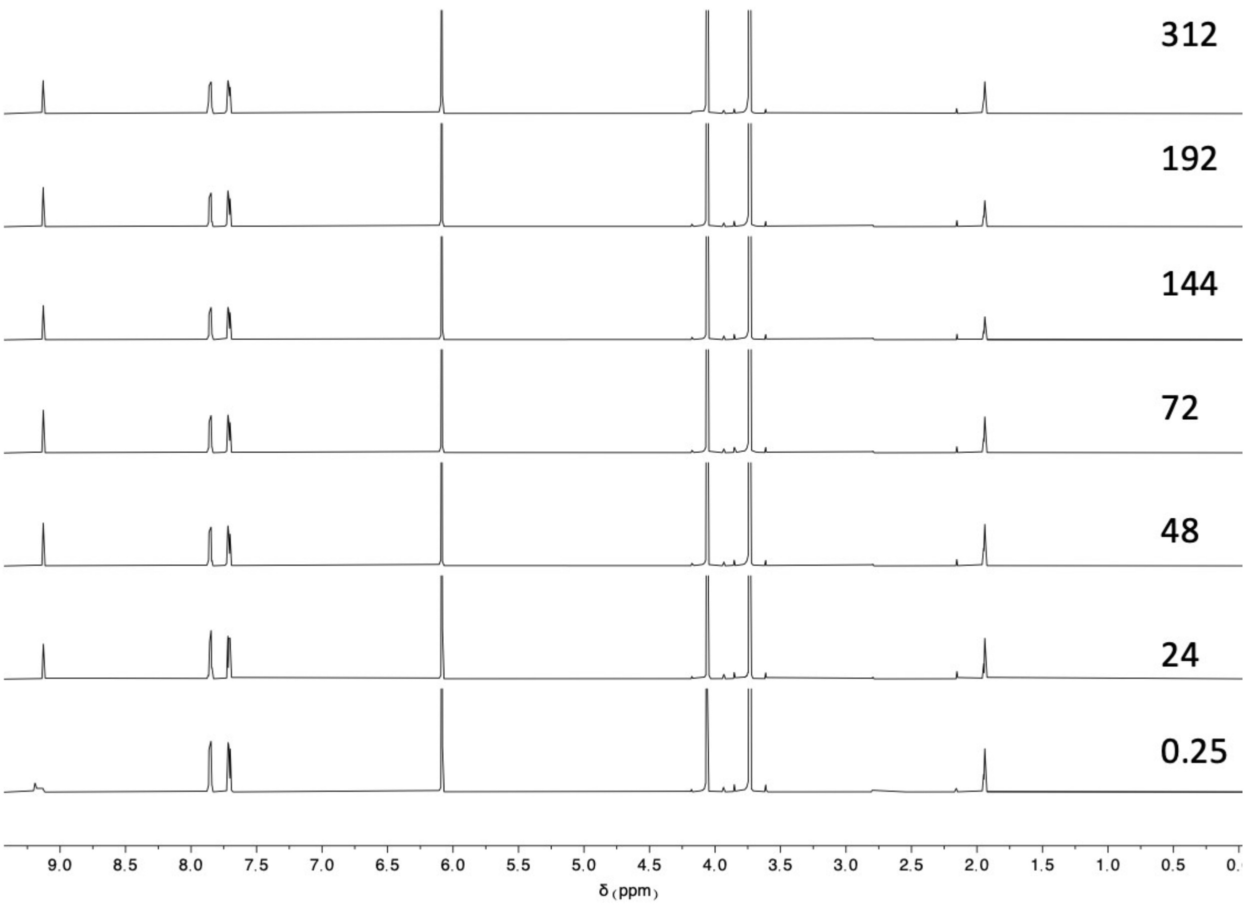

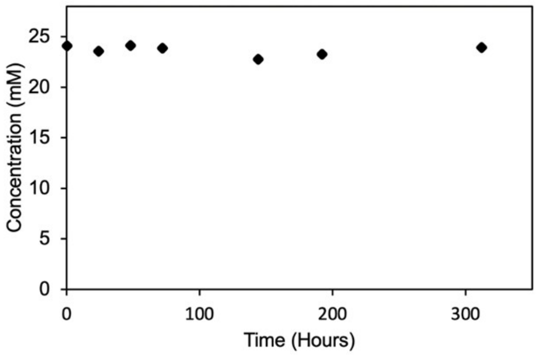

Figure S9: Time dependent ^{1}H NMR spectra of 1,3-dimethyl-1*H*-benzimidazolium iodide in CD_3CN with trimethoxy benzene as an internal standard recorded at 0.25, 24, 48, 72, 144, 192 and 312 hours and corresponding concentration vs time plot.

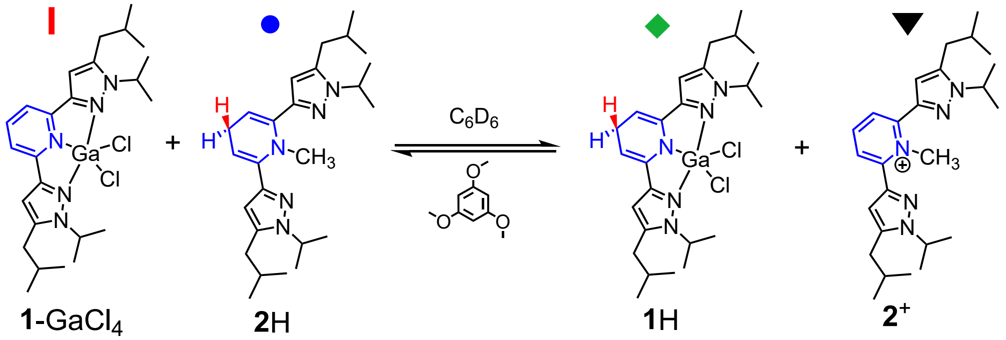

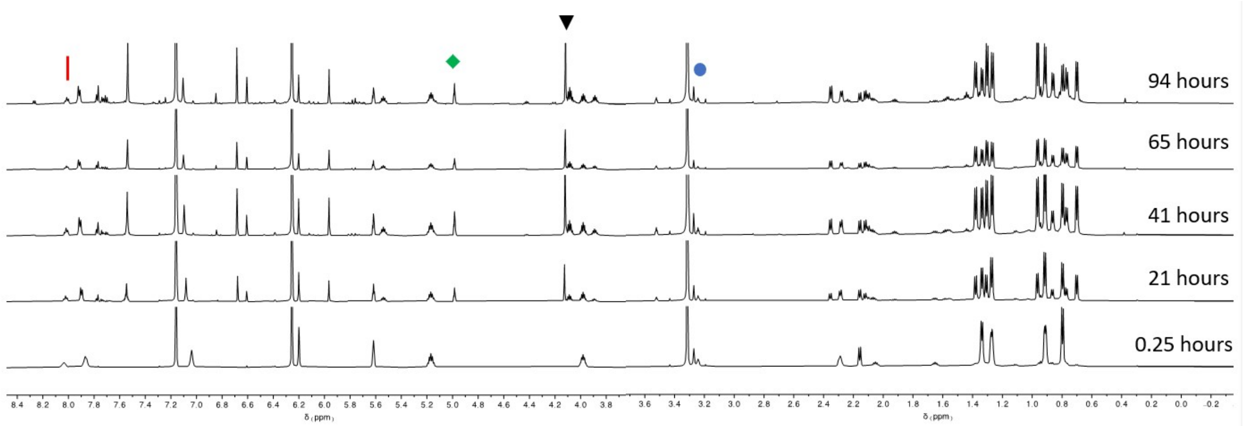

Figure S10. Reaction scheme of **1**-$GaCl_4$ with **2**H in deuterated benzene with trimethoxy benzene as an internal standard (top). Stacked ^{1}H NMR plot of reaction of **1**-$GaCl_4$ and **2**H. Diagnostic peaks for the C_p-*H* signal for **1**-$GaCl_4$ and **1**H, and the N-CH_3 for **2**$^+$ and **2**H species are marked with a red rectangle, greed diamond, black triangle and blue circle respectively. There are two side by side spectra (from -0.2 to 3.6, right and 3.6 to 8.4 ppm, left) scaled such that relevant signals are easily seen. (bottom)

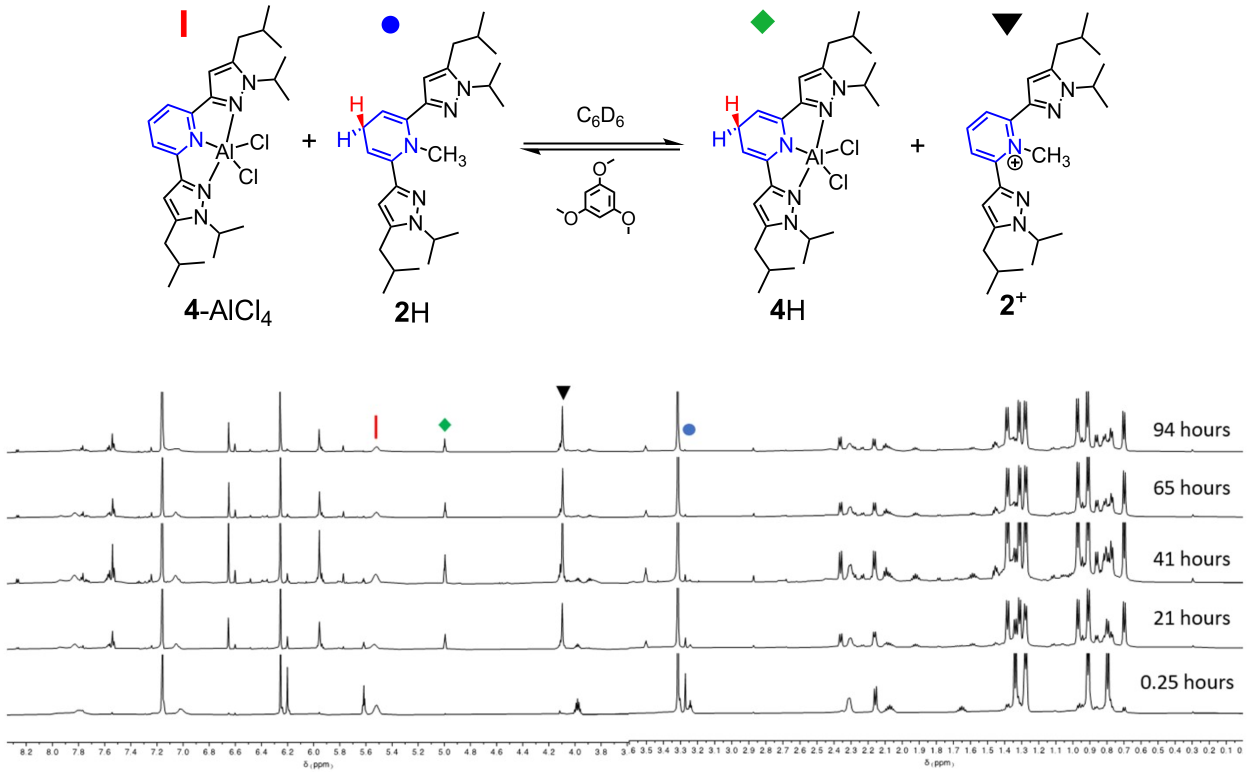

Figure S11. Reaction scheme of **4**-$AlCl_4$ and **2**H in deuterated benzene with trimethoxy benzene as an internal standard (top). Stacked ^{1}H NMR plot of reaction **4**-$AlCl_4$ and **2**H. Diagnostic peaks for the C_p-*H* signal for **4**-$AlCl_4$ and **4**H, and the N-CH_3 signal for $\mathbf{2}^+$ and **2**H species are marked with a red rectangle, greed diamond, black triangle and blue circle respectively. There are two side by side spectra (from -0.2 to 3.6, right and 3.6 to 8.2 ppm, left) scaled such that relevant signals are easily seen (bottom).

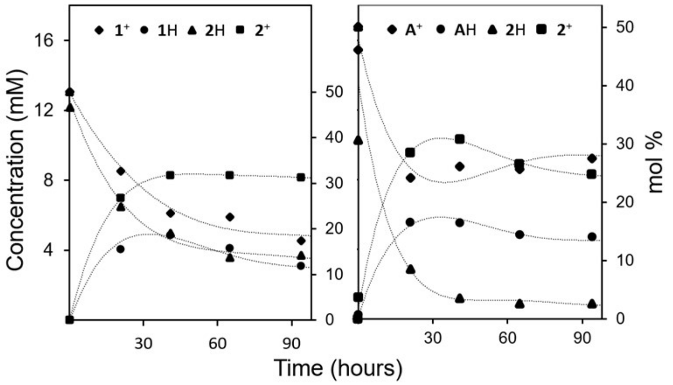

Figure S12: Concentration (mM) and mol % vs time (hours) plotted for: (left) reaction of **1**-$GaCl_4$ with **2**H; (right) reaction of **4**-$AlCl_4$ with **2**H. Lines added to serve as a visual aid but are not fits of the data. Each data point is average of three experiments. Data used to construct these plots shown in Figures S4 and S5.

5. References

[1] T. J. Sherbow, J. C. Fettinger, L. A. Berben, *Inorg. Chem.,* 2017, **56**, 8651-8660.
[2] T. A. Murphree, C. Vorauer, M. Brzoska and M. Guttman, *Anal.Chem.,* 2020, **92**, 9830–9837
[3] SMART Software Users Guide, Version 5.1, Bruker Analytical X-Ray Systems, Inc.; Madison, WI 1999.
[4] SAINT Software Users Guide, Version 7.0, Bruker Analytical X-Ray Systems, Inc.; Madison, WI 1999.
[5] Sheldrick, G. M. SADABS, Version 2.03, Bruker Analytical X-Ray Systems, Inc.; Madison, WI 2000.
[6] Sheldrick, G. M. SHELXTL Version 6.12, Bruker Analytical X-Ray Systems, Inc.; Madison, WI 1999.
[7]A. W. Addison, T. N. Rao, J. Reedijk, J. van Rijin and G. C. Verschoor, *J. Chem. Soc. Dalton Trans*., 1984, **7**, 1349-1356.
